# Pseudodementia in Patients with Unipolar and Bipolar Disorders: A Case Series and Literature Review

**DOI:** 10.3390/jcm13061763

**Published:** 2024-03-19

**Authors:** Camilla Elefante, Giulio Emilio Brancati, Donatella Acierno, Gabriele Pistolesi, Sara Ricciardulli, Francesco Weiss, Francesca Romeo, Lorenzo Lattanzi, Icro Maremmani, Giulio Perugi

**Affiliations:** 1 Psychiatry Unit, Department of Clinical and Experimental Medicine, University of Pisa, 56126 Pisa, Italy; camilla.elefante@phd.unipi.it (C.E.); giuliobrancati@gmail.com (G.E.B.); diletitta@gmail.com (D.A.); gabrielepistolesi@gmail.com (G.P.); sara.ricciardulli@yahoo.com (S.R.); francesco.weiss93@gmail.com (F.W.); giulio.perugi@med.unipi.it (G.P.); 2 Psychiatric Unit, NHS Cuneo Unit 1, 12100 Cuneo, Italy; fraromeo154@gmail.com; 3 Psychiatric Unit, Santa Chiara University Hospital, 56126 Pisa, Italy; llattanzi@blu.it; 4 Saint Camillus International University of Health and Medical Sciences (UniCamillus), 00131 Rome, Italy; 5 G. De Lisio Institute of Behavioral Sciences, 56127 Pisa, Italy

**Keywords:** pseudodementia, depressive pseudodementia, manic pseudodementia, bipolar disorder, bipolar spectrum, mood spectrum, predementia, reversible dementia, cognitive reserve, neurodegeneration

## Abstract

Even though pseudodementia has been historically linked to depression, other psychiatric conditions may cause reversible cognitive alterations. The purpose of this study is to improve our understanding of pseudodementia occurring throughout the entire bipolar spectrum. A systematic review was conducted according to PRISMA guidelines. PubMed, Scopus, and Web of Science databases were searched up to March 2023. Fifteen articles on patients with pseudodementia and bipolar disorder (BD), mania, hypomania, or mixed depression have been included. Moreover, seven female patients with mood disorders diagnosed with pseudodementia have been described. According to our research, pseudodementia in patients with BD mostly occurs during a depressive episode. However, pseudodementia has also been observed in the context of manic and mixed states. Psychomotor and psychotic symptoms were commonly associated. The most typical cognitive impairments were disorientation, inattention, and short-term memory deficits. Alterations in neuroimaging were frequently observed. Electroconvulsive therapy and lithium, either alone or in combination with antipsychotics, resulted in the most widely used therapies. Cognitive decline may occur in a substantial proportion of patients. Since pseudodementia can manifest along the entire mood spectrum, it should be taken into consideration as a possible diagnosis in BD patients showing cognitive deficits during manic, mixed, and depressive states.

## 1. Introduction

Pseudodementia is a term indicating cognitive deficits caused by psychiatric conditions that mimic neurodegenerative diseases. Despite being mostly described in elderly patients with depression, the term pseudodementia can also refer to cognitive alterations caused by other mental disorders. As opposed to what occurs in dementia, cognitive impairments in pseudodementia are reversible upon resolution of psychiatric symptoms [1].

The concept of pseudodementia was first introduced in 1961 when the British psychiatrist Leslie Kiloh reported a case series of 10 patients with apparent dementia due to psychiatric disorders [2]. As stated by Kiloh, in those cases, not only depression but also mania, psychosis, catatonia, conversion disorder, and malingering may impair memory and produce clinical pictures that can be misinterpreted as dementia [2]. Clinicians should be aware of this condition since it severely affects both functional and cognitive skills but is largely reversible [1].

According to Wells criteria, patients with pseudodementia were more likely to have an abrupt onset, along with excessive dependency, subjective distress and awareness of deficit, global deficit, poor effort and cooperation, incongruent cognitive and behavioral performance, and absence of nocturnal worsening, compared to patients with dementia [3].

The frequency of pseudodementia in community-dwelling elderly people has been reported to be less than 1% [4]. A higher rate of pseudodementia, however, has been found in patients who require a clinical assessment for cognitive decline, especially those under the age of 65 [5,6].

Studies looking at the outcome of pseudodementia have produced contradictory findings [7]. According to some authors [8], patients with pseudodementia have a high probability of developing dementia over time, but according to other authors, they have a good prognosis [9,10]. A systematic review of longitudinal studies concluded that the development of irreversible dementia is more common when pseudodementia occurs late in life. Accordingly, dementia will only occur in 2% of individuals who have pseudodementia before the age of 73, while 60% of those developing the disease after that age will have irreversible dementia [7].

Since pseudodementia could ultimately progress to dementia, it was considered, at some point, a meaningless and misleading concept [11]. Another objection to the construct validity of pseudodementia in the context of depression involves the close relationship between late-onset mood disorders and dementia. Late-life depressive disorders, in fact, may often be associated with or precede dementia [12]. In particular, individuals who develop depression for the first time in late life may be at high risk of developing dementia over time [13,14]. Neurobiological research, in turn, suggests that late-life depression may present cerebrospinal fluid biomarkers similar to those of Alzheimer’s disease, supporting the idea that the two disorders share, at least in part, the same pathogenic processes [15]. However, these observations do not undermine the descriptive validity of the concept of pseudodementia but emphasize that in this condition, the observable cognitive deficits are, at least for a period of time, reversible once the underlying mental disorder is successfully treated [16].

Although pseudodementia seems to result from a variety of psychiatric disorders, the large majority of studies have primarily focused on depressive pseudodementia in patients with single-episode or recurrent major depressive disorder (MDD). Due to the small number of studies, case reports, and series, little is known about the prevalence, symptomatology, management, and outcome of pseudodementia caused by other psychiatric disturbances. A number of cases of pseudodementia in patients with bipolar disorder (BD) have been reported in the literature. It is likely that pseudodementia in the context of BD was overlooked due to the underdiagnosis of BD, particularly BD type 2. As a matter of fact, up to 40% of patients with mood disorders initially received an incorrect diagnosis of unipolar depressive disorder; these patients were later more appropriately classified as having BD [17,18]. In particular, BD seems to be underdiagnosed in old age. In fact, it was historically believed that mania decreased with age [19,20]. Epidemiological studies, however, have lately revealed that the prevalence of BD in older patients is comparable to that in adults [21,22], and, depending on the setting, it may be even greater [23,24]. Notably, an association has been found between neurodegenerative conditions and BD. The latter, indeed, often precedes, overlaps, or occurs together with neurodegenerative disorders [25,26,27].

In order to summarize the existing knowledge about the psychopathology, treatment, and outcome of pseudodementia throughout the entire bipolar spectrum, we systematically reviewed the available literature on bipolar and mixed depression, as well as manic and hypomanic states. We have also described a series of patients with pseudodementia and mood disorders, including unipolar and BD, who were referred to our Psychiatric Department for diagnosis and treatment.

## 2. Materials and Methods

### 2.1. Literature Review

#### 2.1.1. Search

A systematic review of the literature was conducted, and the Preferred Reporting Items for Systematic Reviews and Meta-Analyses (PRISMA) guidelines were used to describe procedures and results [28]. PubMed, Scopus, and Web of Science bibliographic databases were searched from their date of inception to March 2023. Reference lists of included studies were also carefully searched for relevant citations. The research team discussed and reviewed the results of an initial scoping search. We developed a strategy using two groups of search terms. These were ‘pseudodementia’ or ‘reversible dementia’ or ‘pseudodementia’ (group 1) AND ‘mania’ or ‘manic’ or ‘hypomania’ or ‘hypomanic’ or ‘bipolar disorder’ or ‘mixed depression’ or ‘bipolarity’ (group 2). Terms were adapted as necessary for each database. The results were downloaded into Mendeley software version 1.19.8.

#### 2.1.2. Eligibility Criteria

The search included reviews and original studies in the English language. Only original studies, including case reports and case series, were eligible for our review. If a previous review was found, we searched the reference list to identify and retrieve the primary studies. No restrictions on study design or group comparisons were applied. In order to be included in our review, study participants or subgroups of participants should have been diagnosed with pseudodementia and BD, mania, hypomania, or mixed depression. No restrictions for age nor for age at onset, either of dementia or BD, were applied. All studies on clinical features, course, and treatment of patients who were cross-sectionally or longitudinally diagnosed with BD and pseudodementia were included.

#### 2.1.3. Abstract Screening and Study Selection

A total of 163 abstracts were retrieved using our search strategy, of which 44 were removed as duplicates. Thus, 119 abstracts were screened. If a title appeared potentially eligible but no abstract was available, the full-text article was retrieved. Two researchers (CE and SR) scanned all titles and abstracts to identify relevant articles for full-text retrieval. Disagreements were resolved by discussion after consulting a third reviewer (GEB). A total of 104 records were excluded based solely on title or abstract. Unfortunately, it was not possible to retrieve the full text of Allen, 1982 [29]. A total of 14 full-text articles were thus thoroughly assessed for eligibility. Three additional records were identified through other sources (citations in reference lists of screened papers and reviews) and assessed for eligibility [30,31,32]. Two articles focusing on reversible dementia, respectively, due to lithium intoxication and vitamin B12 and folate deficiency, were excluded [33,34]. A total of 15 studies were finally included in the systematic review.

### 2.2. Case Series

We retrospectively selected the clinical charts of seven female patients diagnosed with pseudodementia attending our Psychiatry Department. The cases of inpatients have been retrieved from a pool of subjects admitted to a female-only ward, whereas those of outpatients have been retrieved from a sample of subjects followed up at our psychogeriatric service. The ages of the patients described ranged between 58 and 81 years old. Different mood disorders were represented, ranging from single-episode MDD in one case to recurrent MDD in two cases, BD type 2 in three cases, and BD type 1 in one case.

Pseudodementia is defined as a psychiatric disorder that presents as a neurodegenerative disease that is largely reversible after resolution or treatment of the underlying psychiatric illness [1,2], but there are no official diagnostic criteria for its diagnosis. We included in our sample patients who met Diagnostic and Statistical Manual of Mental Disorders, Fifth Edition (DSM-5) criteria for both mood disorders and major neurocognitive disorders at admission in our inpatient or outpatient clinic but who no longer met the criteria for major neurocognitive disorders following resolution of mood symptoms.

## 3. Results

### 3.1. Literature Review

Based on the results of our systematic search, pseudodementia has been reported in 30 patients with BD. Most patients were women (20 of 30, 67%), with ages ranging between 45 and 79 years old, with about one-half of subjects being 65 years old or older. In approximately two-thirds of patients, pseudodementia features occurred during a depressive episode (19 of 30, 63%) [9,30,32,35,36,37]. However, pseudodementia has also been described in seven patients with manic or mixed episodes [9,38,39,40,41,42,43] and in one patient diagnosed with a depressive mixed state [44]. Severe impairment of attention and short-term memory during a whole manic-depressive cycle was reported in one patient [31], while another with pseudodementia features during mixed mania also experienced depressive pseudodementia during follow-up [42]. Finally, intermittent pseudodementia has been described in one patient with a long-lasting history of rapid and continuous manic-depressive cycles [40].

Psychomotor disturbances and psychotic symptoms were common, being respectively reported in twelve [31,32,36,37,38,39,40,42,43,44] and seven cases [30,32,35,39,43,44]. Most cases presented with disorientation, attention, and short-term memory impairments. Fluctuations of affective and/or cognitive symptoms were sometimes noted [39,43,44], making the differential diagnosis of delirious mania difficult. Non-specific electroencephalogram (EEG) abnormalities were found in about one-third of patients (8 of 22, 36%) [9,32,36,37,38,40,41]. Cortical atrophy, ventricles, or sulcal enlargement were observed in almost half of subjects with different neuroimaging techniques (7 of 16, 44%) [31,35,37,38,40,41,43]. Two patients showed a non-specific pattern of hypometabolism in the parietal and temporal regions at nuclear medicine imaging [35,44].

In most cases, especially those with depressive episodes, symptoms lasted for 6 months or more. Importantly, cognitive function was restored even after years in two cases [31,35]. Electroconvulsive therapy (ECT) and lithium salts, either in monotherapy or combined with antipsychotics, were the most commonly reported treatments. ECT was used in more than half of patients (11 of 22, 50%) [29,32,36], including one patient who had been previously treated effectively with a combination of venlafaxine, mirtazapine, and aripiprazole but repeatedly relapsed [44] and one patient who had not improved after being treated with antidepressants, antipsychotics, and even lithium [36]. While most of these cases were diagnosed with major depression with or without mixed features, lithium was preferentially used in manic patients (8 of 22, 36%) [31,38,39,41,42,45,46]. In addition, one patient with depressive pseudodementia was effectively treated with nortriptyline [35]. Lastly, a patient who was taking many medications, including lithium, had an improvement in his cognitive and neurological state after a significant decrease in the medications’ dose [37].

Follow-up was available only in 15 cases, with a variable duration ranging between one and twelve years. Overall, about one-fifth of patients (4 of 15, 27%) showed cognitive decline: two subjects were finally diagnosed with dementia [30], one failed to regain his premorbid level of cognitive ability [37], while the patient with rapid-cycling BD had a progressive decrease in his performances at cognitive testing [40]. The features of the cases that have been collected from the literature are summarized in Table 1.

### 3.2. Case Series

#### 3.2.1. Case 1: Late-Onset, Single-Episode Depressive Pseudodementia

F.S. was a 74-year-old woman admitted to our inpatient psychiatric clinic for melancholic depression twice within a short period of time. Her family history was negative for mental illnesses and neurodegenerative diseases. She had diabetes mellitus type 2, hypertension, osteoporosis, and a grade I cystocele. She had a 5-year education. She had never experienced affective disturbances until the age of 72, when, after a loss event, she began to develop depressive symptoms, including low mood, generalized anxiety, somatic complaints, and hyporexia. Additionally, she progressively started to experience difficulties taking care of her personal needs, and her overall functioning became scarce. The patient’s mental health deteriorated over time. She became increasingly anxious, restless, and oppositional. Her content of thought was polarized on hypochondriac ideas: she insistently reported somatic complaints at the gastric level, for which she made multiple visits to the emergency room. Despite the fact that no gastrointestinal abnormalities were found, she was totally convinced that something was wrong and that the doctors might harm her. She was diagnosed with depression, and cognitive decline was suspected by a psychiatric expert who provided consultation in the emergency room and indicated a psychiatric follow-up. She was initially prescribed sertraline and subsequently changed to vortioxetine and mirtazapine. Since agitation appeared and anxiety worsened, olanzapine and alprazolam were added to antidepressants without success. Several combinations of second-generation antipsychotics and antidepressants were subsequently prescribed without clinical benefit. She had a progressive deterioration in hygiene and physical conditions, and hospitalization at our clinic was recommended when she was 74.

At admission, she was reluctant to be interviewed. However, ruined ideas and somatic delusions emerged. She was convinced she could not assimilate foods and was no longer able to urinate and walk. Clinophilia, insomnia, hyporexia, and psychomotor retardation alternated with restlessness were also evident. A brain computed tomography (CT) scan revealed areas of gliosis resulting from chronic vascular encephalopathy. Although she was incapable of doing simple activities like bathing and clothing herself, she was oriented in time and space. She scored 26/30 on the Mini-Mental State Examination (MMSE). Pregabalin, nortriptyline, and trimipramine were introduced, with some improvement in mood. She was discharged, but a week later, she experienced an aggravation of the index symptomatology, necessitating her readmission. Thirteen brief-pulse bitemporal ECT sessions were delivered on a twice-a-week schedule, with marked improvement in mood and functioning. ECT was administered using the brief pulse stimulator Mecta 5000Q (Mecta Corporation, Lake Oswego, OR, USA). Parameters included a pulse width of 1.0, a duration ranging from 2.0 s to 3.0 s, a frequency ranging from 60 Hz to 90 Hz, and a current of 0.8 A. The initial stimulus dosage was based on the “half-age” stimulation strategy for ECT dosing [47]. During the ECT course, the stimulus dosage was adjusted to maintain a seizure duration of at least 25 s. Anesthesia was induced with intravenous thiopental (2–4 mg/kg), and muscle relaxation was assured with succinylcholine (0.5–1 mg/kg). Motor and EEG seizure durations were monitored. She resumed normal gait and speech; her gastrointestinal and urinary complaints disappeared; and she did not require assistance to carry out the activities of daily living. The MMSE score was 27/30 at discharge. Lithium carbonate, pregabalin, and nortriptyline were prescribed.

After discharge, she returned to live alone for a few weeks. She also started riding her bicycle again. In the following 4 years, the patient stayed well without changes to pharmacological therapy. She had no further affective episodes or cognitive impairment and maintained satisfactory overall functioning.

#### 3.2.2. Case 2: Depressive Pseudodementia in a Patient with Late-Onset Recurrent MDD

S.D.P. was a 76-year-old woman admitted to our outpatient psychiatric clinic for a depressive episode. The family history was positive for mood disorders and Alzheimer’s disease. She had a 5-year education. She had experienced recurrent major depressive episodes starting from the age of 67, at which point, following a stressful life event, she developed a depressed mood, anxiety, insomnia, and hyporexia. From that point on, she was treated at her local mental health service until she was 76. Amitriptyline, perphenazine, and delorazepam were prescribed for her first depressive episode with a resolution of symptoms. In the following three years, she had two depressive relapses for which she was prescribed antidepressant treatment, first with mirtazapine and, subsequently, with nortriptyline. Between 71 and 76 years old, she stayed euthymic on nortriptyline (50 mg/day).

At the age of 76, the patient abruptly stopped her maintenance therapy due to the commercial unavailability of nortriptyline in Italy and had a depressive relapse. She experienced a depressed mood, internal tension, emotional lability, terminal insomnia, panic attacks with dyspnea, dizziness, and gastric somatizations. Additionally, memory deficits appeared, and she struggled with doing regular tasks. In particular, she complained about having trouble locating recently used items. She was worried about developing a neurodegenerative disease, so she voluntarily consulted the neurological unit and underwent a series of neuropsychological tests, which revealed alterations in working memory and attention. Her MMSE score was 25/30. A brain CT scan showed modest hypodensities of periventricular white matter. She was prescribed paroxetine (20 mg/day), amitriptyline (50 mg/day), trimipramine (20 mg/day), and pregabalin (150 mg/day) with a reduction in anxiety and panic attacks, improvement of energy and mood, and regularization of sleep over a few months. After approximately 5 months, neuropsychological tests normalized, except for divided attention, so that she received the diagnosis of depressive pseudodementia. She regained her usual level of functioning and was capable of doing all of the housekeeping.

In the following years, however, despite no affective relapses, short-term memory deficits gradually appeared. Three years after depressive pseudodementia, she was evaluated again by a neurologist, and brain magnetic resonance imaging (MRI) was recommended. Neuroimaging revealed a reduction in the volume of the frontal lobe, temporal lobe, and hippocampus. The MMSE score was 20/30. She was diagnosed with mild cognitive impairment, and donepezil was prescribed. Nowadays, at the age of 81, she needs supervision to go out and shop, take medicine, and cook, but she is able to maintain personal care and hygiene and continue to practice some hobbies like embroidery.

#### 3.2.3. Case 3: Depressive Pseudodementia in a Patient with Recurrent MDD and OCD Comorbidity

L.B. was a 60-year-old woman admitted to our inpatient psychiatric clinic for a depressive episode with comorbid obsessive–compulsive disorder (OCD). The family history was positive for ictus. She had a history of recurrent MDD with comorbid OCD, and she was also affected by hypertension. She completed 13 years of schooling. At the age of 30, after a loss event, she developed her first depressive episode with co-occurring obsessive thoughts and compulsions. Successful treatment with fluvoxamine and benzodiazepines had been introduced at that time and maintained for subsequent years with only mild fluctuations of mood and obsessive–compulsive symptoms.

At the age of 56, the patient had a major recurrence of depressive symptoms associated with increased anxiety. Progressive worsening of the clinical picture occurred with the re-appearance of obsessions and checking rituals. Several antidepressants were prescribed in sequence by her psychiatrist, including selective serotonin reuptake inhibitors (SSRIs) (paroxetine up to 20 mg/day, citalopram up to 20 mg/day, fluvoxamine up to 200 mg/day), and clomipramine (up to 150 mg/day), without remission of symptoms. Subsequently, valproic acid (up to 300 mg/day) and benzodiazepines were introduced, with no significant improvements. Executive deficits and functional impairments gradually emerged, and at the age of 58, the patient was forced to leave her job at the cadastral office, where she had been working for decades. She also became socially isolated and spent a lot of time doing rituals like inspecting and measuring drawings of old buildings, checking the garbage to make sure nothing priceless was thrown away, and checking that her underwear was not dirty. At the age of 59, she consulted another psychiatrist, who prescribed various treatment regimens with mood stabilizers (lithium sulfate and valproic acid), antidepressants (fluvoxamine, paroxetine, sertraline, and amitriptyline), antipsychotics (risperidone, perphenazine, and paliperidone), anticholinergics (biperiden), and benzodiazepines. The clinical picture worsened with the appearance of psychomotor changes, including both retardation and agitation, and a reduction in personal autonomy. The patient was visited again by her first psychiatrist, who prescribed valproic acid (up to 600 mg/day), pregabalin (up to 75 mg/day), fluvoxamine (up to 300 mg/day), sertraline (up to 100 mg/day), and delorazepam without sustained clinical improvements. During an episode of agitation, her husband brought her to the emergency room, where she was prescribed valproic acid (1500 mg/day), lurasidone (18.5 mg/day), and delorazepam (4 mg/day). After this therapeutic change, agitation and restlessness increased.

At the age of 60, hospitalization at our clinic was recommended for the persistence of symptoms. At admission, the patient was unable to stop talking and repeated the same questions all the time in search of reassurance. Verbal perseveration and compulsive behaviors were observed. Psychomotor retardation alternated with agitation.

Disorientation in time, severe attention deficits, recall difficulties, and word-finding deficits clearly emerged on examination. She scored 21/30 at MMSE. With the neurologist’s advice, the patient underwent a brain MRI that showed a slight enlargement of periencephalic liquoral spaces at the vertex, two hyperintense areas in the right corona radiata, and one in the medial orbital cortex. Since she had extrapyramidal signs, lurasidone was discontinued, and ^123^I-iofluopane single-photon emission computerized tomography (SPECT) was performed, with negative results. She also underwent ^18^F-fluoro-2-deoxy-D-glucose (FDG)-positron emission tomography (PET) that revealed mild global hypometabolism in the cortex, mostly in the prefrontal and medial frontal regions bilaterally. Because of the concomitant acute psychiatric symptomatology, the neurological findings were not considered to be of unequivocal interpretation, and neurological follow-up was recommended. Trazodone was introduced to reduce the severity of anxious symptoms, agitation, perseverative behavior, and checking rituals. Pharmacological treatment at discharge included valproic acid (750 mg/day), trazodone (50 mg/day), and delorazepam (1 mg/day).

After the discharge, the patient was regularly visited once a month in our outpatient service, and the therapy was gradually modified. Since she was less anxious and agitated but still had obsessive thoughts and some compulsions, antidepressants (fluvoxamine 300 mg/day and clomipramine 20 mg/day) were gradually reintroduced. Lithium carbonate (450 mg/day) and low-dose quetiapine (125 mg/day) were also introduced in addition to valproic acid. The patient gradually experienced an improvement in psychiatric and cognitive symptoms. While still anxious, checking rituals, psychomotor disturbances, and confusion disappeared. Social and familiar functioning progressively improved. Residual psychiatric symptoms, including anhedonia, anxiety, and some obsessions, still persisted two years after discharge, in spite of treatment adjustments. She spent the majority of her time at home and went out one time a week to do shopping, but only if accompanied by a family member. However, she was able to do housework and play cards with her husband every day but was reluctant to perform more demanding tasks. Despite having a 30/30 MMSE score, she has not yet returned to her antecedent functional level. Both subtle cognitive impairments and residual depressive and obsessive symptoms may negatively affect her functioning.

#### 3.2.4. Case 4: Depressive Pseudodementia in a Patient with BD Type 2

C.M. was a 69-year-old woman admitted to our outpatient psychiatric service for a depressive episode. Her family history was negative for mental illnesses and positive for Parkinson’s disease. She had diabetes and hypertension. She had a 12-year education. Starting at the age of 20, she suffered from panic attacks and depression. At the age of 31, during her second postpartum, she had an episode of hypomania with an increased level of energy and decreased need for sleep that had spontaneously remitted. During the following years, she experienced repeated depressive mood swings with a seasonal pattern, for which she intermittently took a variety of antidepressants, usually stopping them on her own when mood symptoms disappeared, suggesting possible depressive-hypomanic cycles.

At the age of 68, following a stressful life event, the patient had a depressive episode that was more severe than the previous ones. For this episode, which was characterized by lack of energy, low mood, loss of interest, hopelessness, anxiety, and agoraphobia, she was prescribed paroxetine (20 mg/day), mirtazapine (15 mg/day), perphenazine (2 mg/day), and delorazepam (0.5 mg/day) with initial clinical benefit. After a few months, depressive and anxious symptoms worsened, and psychomotor retardation occurred. Her treating psychiatrist increased paroxetine up to 40 mg/day, mirtazapine up to 30 mg/day, perphenazine up to 6 mg/day, and introduced biperiden (4 mg/day). Hypersomnia, behavioral impairments, cognitive deficits, and several episodes of confusion appeared. Her husband reported that the patient had an episode of confusion during the night in which she had risen from bed and urinated on the ground. When asked about what happened, the patient claimed to not remember anything. She was then referred to the neurological outpatient service. At the neurological examination, she scored 21/30 on the MMSE. Deficits in verbal learning and selective and divided attention were detected in the neuropsychological tests. The brain CT was negative, while the brain MRI showed areas of hyperintensity in the frontal subcortical white matter. FDG-PET did not show metabolic alterations. On neurological advice, the patient was referred to our outpatient service for consultation to exclude iatrogenic conditions.

At the first psychiatric examination in our department, the patient had been acutely ill for nearly a year. She appeared depressed, apathetic, anxious, agoraphobic, and with low energy levels. The thought content was focused on the cognitive difficulties that the patient described with significant emotional participation. Hypomimia, rigidity, and bradykinesia were also present. Paroxetine was reduced to 10 mg/day, and treatment with clomipramine (50 mg/day), trimipramine (20 mg/day), pregabalin (100 mg/day), and low doses of benzodiazepines was introduced, with gradual improvement of symptoms. After 3 months, anxiety and mood considerably improved. Agoraphobia, fatigability, and attention problems still persisted but gradually faded during subsequent months.

After 6 months from the first consultation at our service, the improvement in affective and cognitive symptoms was evident. The patient had recovered her energy levels, no longer had psychomotor retardation, and was able to go on vacation with her husband. She went to the gym and attended English courses. At her second neurological evaluation, she scored 25/30 on the MMSE, and neuropsychological tests were within the normal range. At one year of follow-up, she scored 30/30 on the MMSE. During the following 3 years, a hypomanic episode occurred, for which clomipramine was reduced to 10 mg/day and pregabalin was substituted with valproic acid (450 mg/day). An additional anxious–depressive episode also occurred, with no changes in cognitive functioning. Lithium carbonate (300 mg/day) was finally added. Despite mood swings, no memory deterioration occurred in the 4 years following pseudodementia, with the patient never scoring below 29/30 on MMSE.

#### 3.2.5. Case 5: Depressive Pseudodementia with Psychosis in a Patient with BD Type 2 and OCD Comorbidity

A.B. was a 58-year-old woman admitted to our inpatient psychiatric clinic for a severe psychotic depressive episode. Her family history was positive for mood disorders and negative for neurodegenerative diseases. She had hypertension, hypercholesterolemia, nephrolithiasis, and obesity. She had a 9-year education. She had a history of BD type 2 with comorbid OCD. At the age of 14, she developed her first depressive episode with low mood, anxiety, cleaning obsessions, compulsive handwashing, and severe impairment of functioning, leading the patient to abandon her studies. At that time, clomipramine was prescribed with a remission of symptoms. At the age of 22, a second depressive episode with similar features occurred, forcing the patient to quit her job. In the following years, she experienced periods of euthymia alternating with mood swings of both polarities. While fear of contamination and cleaning rituals characterized depressive episodes, obsessive–compulsive symptoms disappeared during hypomanic episodes that were instead characterized by elated mood, increased energy, talkativeness, and goal-directed activities. She had been thus treated with a combination of mood stabilizers, including lithium carbonate, and, depending on the affective phase, antidepressants or low-dose antipsychotics.

At the age of 58, she started complaining about mouth pain with co-occurring depressive symptoms. Somatic complaints gradually assumed the features of a somatic-type delusional disorder. Although only a small injury was found at the medical examination, the patient began to reduce her food and beverage intake. Psychomotor retardation and mental confusion occurred, leading to admission to the local psychiatric hospital. Acute renal failure likely due to high lithium blood levels caused by dehydration was diagnosed, lithium was stopped, and hemodialysis was started. After renal impairment had recovered, lamotrigine (100 mg/day) and lorazepam were gradually introduced, and the patient was discharged. Since depressive mood, bradykinesia, anxiety, somatic delusion, guilt ideas, cognitive alterations, and refusal to eat and drink persisted, another psychiatric hospitalization was necessary after a short time. During her second hospitalization, she was treated with several combinations of mood stabilizers (valproic acid, lamotrigine), selective serotonin reuptake inhibitors, antipsychotics (haloperidol, quetiapine, cariprazine, quetiapine), and lorazepam. Psychiatric symptoms worsened, and she refused to feed, making necessary the placement of a nasogastric tube and transfer to our psychiatric inpatient clinic.

At admission, she had a psychomotor slowdown that alternated with episodes of agitation and restlessness, especially during the night. She was disoriented in time and showed marked difficulties in performing simple tasks, such as getting dressed. She was convinced she had urinary incontinence and repeatedly checked to see if she had urinated in the diaper. She perseverantly repeated that she was unable to manage her intimate hygiene. A MMSE score of 24/30 was obtained. A brain CT scan ruled out any acute neurological issues. Hematologic, biochemical, metabolic, inflammatory, and thyroid blood tests yielded unremarkable results. She was treated with a combination of valproic acid, tricyclic antidepressants (clomipramine and amitriptyline), and typical and atypical antipsychotics without clinical benefit. Four weeks after admission, the medical team decided to treat her with ECT. Eight brief-pulse bitemporal ECT sessions were delivered on a twice-a-week schedule. ECT was administered using a brief pulse stimulator, the Mecta 5000Q (Mecta Corporation, Lake Oswego, OR, USA). Parameters included a pulse width of 1.0, a duration ranging from 2.0 s to 3.0 s, a frequency ranging from 60 Hz to 90 Hz, and a current of 0.8 A. The initial stimulus dosage was based on the “half-age” stimulation strategy for ECT dosing [46]. During the ECT course, the stimulus dosage was adjusted to maintain a seizure duration of at least 25 s. Anesthesia was induced with intravenous thiopental (2–4 mg/kg), and muscle relaxation was assured with succinylcholine (0.5–1 mg/kg). Motor and EEG seizure durations were monitored. Clinical improvement started after the fifth session. At the end of treatment, the patient appeared oriented in time and space, euthymic, and without anxious or obsessive symptoms. She was autonomous in personal care and hygiene. The MMSE score improved to 30/30. After the nephrologist’s approval, lithium salts were reintroduced, and she was discharged with a combination of mood stabilizers (lithium salts 300 mg/day and valproic acid 750 mg/day) and tricyclic antidepressants (clomipramine 50 mg/day and amitriptyline 50 mg/day).

After one month of discharge, she was again able to drive. After six months, she could perform her daily duties again. During the subsequent period, she experienced brief subclinical mood swings and thoughts of contamination, which necessitated therapy adjustments such as the introduction of low doses of antipsychotics (quetiapine 150 mg/day) in addition to antidepressants (trimipramine) and mood stabilizers (lithium salts and oxcarbazepine), but familiarity and social functioning remained adequate. No cognitive deficits occurred. She continued to perform well and maintained an unaltered cognitive level for one year and a half of follow-up.

#### 3.2.6. Case 6: Recurrent Depressive Pseudodementia in a Patient with BD Type 2

M.G. was an 81-year-old woman who was admitted twice to our inpatient psychiatric clinic for two episodes of depression. Her family history was positive for mood disorders and suicide attempts and negative for neurodegenerative diseases. She was affected by dyslipidemia and thrombocytosis. She had a 5-year education. The onset of her psychiatric disorder dates back to the age of 24, when, in the first postpartum, she developed a first depressive episode characterized by anxiety, insomnia, and feelings of guilt or inadequacy about the newborn, which culminated in a suicide attempt. In the following years, she had several depressive recurrences alternating with hypomanic phases. During the depression, she usually showed volitional inhibition, apathy, anhedonia, anxiety, feelings of hopelessness, obsessive thoughts with compulsions of order and cleanliness, insomnia, and suicidal ideation. Hypomanic phases were characterized instead by a euphoric or irritable mood, a high level of energy, increased activity and projectuality, and prodigality. She had been followed by several psychiatrists prescribing different combinations of mood stabilizers and antidepressants or antipsychotics depending on the mood phase, with partial benefit and the maintenance of good global functioning over time.

At the age of 77, she experienced a new hypomanic–depressive cycle. Depression was characterized by holothymic ruminations, anxiety, obsessive–compulsive symptoms, and insomnia. She gradually developed psychomotor retardation, hand resting tremors, memory deficits, concentration deficits, and a need for help and reassurance in daily activities. Given the appearance of mutism, negativism, oppositional behaviors, and refusal to eat, drink, and take oral therapy, she was first hospitalized at our clinic. Given the presence of cognitive deficits and motor symptoms, neuroimaging was prescribed during a neurological consultation. A brain CT scan was negative, while a brain MRI revealed signs of leukoencephalopathy with lacunar lesions in the subcortical white matter and a small meningioma in the anterior frontal right lobe. ^123^I-iofluopane SPECT was negative for nigro-striatal degeneration. FDG-PET showed hypometabolism in the frontotemporal left lobe. During the hospitalization, she was treated with lithium carbonate 150 mg/day, gabapentin 600 mg/day, trimipramine 20 mg/day, and amantadine 100 mg/day, with a progressive improvement of psychiatric and cognitive symptoms in a few weeks. After discharge, the patient recovered her previous functioning and was again able to cook and clean the house. In the following years, she was followed up as an outpatient at our clinic. Amantadine was stopped, and she stayed euthymic, requiring only minor treatment changes.

At the age of 80, after her sister’s death, she had a depressive recurrence with features similar to the previous one. Depressed mood, insomnia, anxiety, obsessive thoughts, checking rituals, pragmatic inhibition, and executive deficits with difficulties doing housework occurred. She was disoriented in time and showed short-term memory deficits. She scored 14/30 at MMSE. Vortioxetine (5 mg/day) and mirtazapine (30 mg/day) were added to her usual therapy. However, her family brought her to the emergency department because she became agitated and aggressive. Antidepressants were suspended by the consultant psychiatrist. Low-dose quetiapine was started, but the patient became sedated and confused. She developed psychomotor retardation, feelings of guilt and incurability, and themes of ruin. She was treated as an outpatient with amantadine, valproic acid, gabapentin, and lithium carbonate. Despite therapeutic changes, her clinical picture did not improve; she became more disoriented, oppositive, agitated, and aggressive, leading to her second admission to our inpatient clinic. During the hospitalization, she was treated with valproic acid (600 mg/day) and mianserin (30 mg/day). Three weeks after her admission, she regained her mood stability and was discharged. At discharge, she was almost completely oriented in space and time and scored 22/30 at the MMSE. Because she had acquired ulcers in her feet during the hospitalization, she lost her ability to walk independently. Once back home, she started using a folding walker. Because of somatic problems and pain, she never recovered from her previous household functioning. Moreover, one year after the discharge, she started to be gradually more apathetic, more anxious, and less autonomous. Her cognitive level was stable, and she scored 22/30 at MMSE, but she needed stimulation to be engaged in daily activities, to perform physiotherapy, and to deambulate. Two and a half years after the discharge, she had a worsening in cognition with a steady decline in her MMSE score to 15/30 and a severe compromise of her global functioning.

#### 3.2.7. Case 7: Manic-Depressive Pseudodementia in a Patient with Late-Onset BD Type 1

L.M. was a 73-year-old woman admitted to our inpatient psychiatric clinic for depression. Her family history was negative for mental illnesses and neurodegenerative disorders. She suffered from hypertension and hypothyroidism. Despite being a very socially anxious and avoidant person, she had never had any affective disorders until the age of 64, when, after her mother had become ill, she developed a manic episode characterized by increased energy, decreased need for sleep, irritability, behavioral disinhibition, physical and verbal aggressiveness, coprolalia, logorrhea, racing thoughts, and visual and auditory hallucinations. This manic state had culminated in an episode of psychomotor agitation that required hospitalization in the Neurology Department of the local hospital. Since she was confused and disoriented, a brain CT and EEG were performed, with negative results. Brain MRI instead showed a modest increase in perivascular spaces without any parenchymal lesions. During the hospitalization, she had been treated with antipsychotics and had developed extrapyramidal signs. ^123^I-iofluopane SPECT, however, was negative for nigrostriatal degeneration. She had been diagnosed with cognitive decline at discharge, and treatment with benzodiazepines and acetylcholinesterase inhibitors had been prescribed.

During the following year, she suffered from several manic-depressive cycles. Manic episodes were characterized by features similar to the index episode, and depressive ones by low mood, apathy, clinophilia, psychomotor retardation, oppositional behavior, hyporexia, and negativism. She had several relapses that required hospitalization in the local psychiatric ward. She did not achieve mood stability despite therapy changes, which included varying combinations of benzodiazepines and antidepressants or antipsychotics, depending on the phases. FDG-PET had been performed, revealing widespread cerebral cortical hypometabolism, involving most prominently the frontotemporal lobes and the left posterior parietal lobe.

At the age of 65, she had a manic relapse with irritability and aggressiveness, followed by a severe depressive-mixed episode characterized by psychomotor retardation, negativism, and oppositional behaviors, for which she was hospitalized. Since she had shown a poor response to the drug treatments previously prescribed, she was given a course of ECT in a private hospital with a favorable initial response. At discharge, she was prescribed olanzapine 10 mg/day and haloperidol 3 mg/day, but after a period of eight months, a depressive switch occurred. She was hospitalized again in a tertiary care university hospital and underwent another ECT course. After 5 ECT sessions, the patient developed a manic episode characterized by an elated mood, reduced need for sleep, logorrhea, coprolalia, and behavioral disinhibition. For these symptoms, she was treated with a therapy based on valproic acid (600 mg/day), sertraline (50 mg/day), quetiapine (300 mg/day), and benzodiazepines. In the following 4 years, she stayed euthymic on this therapy. Moreover, she regained her previous level of functioning, being able to live alone and do her housework.

At the age of 71, she had another manic episode with increased energy, irritability, and physical and verbal aggressiveness, followed by mixed depression, for which she was referred to our inpatient psychiatric clinic. At the admission, she was not accessible for a psychiatric interview. She showed psychomotor retardation, clinophilia, oppositional and disinhibited behaviors, coprolalia, negativism, verbal aggression, and refusal to eat, drink, and take medications. Disorientation, confusion, and gross memory deficits were also present. The speech was confabulating, coprolalic, and perseverative. During hospitalization, brain MRI was repeated and showed moderate atrophy, especially in the left lobe, with the consensual expansion of the ventricular system and liquoral spaces, mostly in the temporal and fronto-opercular cortex. A second FDG-PET showed moderate hypometabolism in the dorsolateral parietal, temporolateral, and temporopolar regions of the left hemisphere. She was prescribed lithium carbonate (450 mg/day), valproic acid (1000 mg/day), clozapine (75 mg/day), and memantine (10 mg/day), with gradual improvement of mood and cognitive symptoms.

After the discharge, she needed some help with activities of daily living, so her sister decided to move into her home. Three months later, she conducted a neuropsychological evaluation, showing only a deficit in a visuomotor speed test. She scored average on the remaining tests and 26/30 at the MMSE. She gradually regained her autonomy in daily life activities, and after 4 months of discharge, she was able to live alone. Memantine was withdrawn, valproic acid was reduced to 600 mg/day, and clozapine was reduced to 50 mg/day without any recurrence. After about a year of discharge, the patient was again able to take the bus on her own and go to the gym. Although she complained of some short-term memory deficits, she scored 29/30 at the MMSE. In the two years following hospitalization, she had neither mood relapses nor significant cognitive alterations.

In our case series, ages ranged from 58 to 81, with a significant proportion of them being 65 years of age or older. Our sample is entirely comprised of female subjects. The onset of mood disorders was in late life in two cases with MDD, respectively, at 67 and 72 years old, and in the case of BD type 1, at 64 years old. Three out of four BD patients from our series of cases showed pseudodementia features during depressive episodes, while the remaining BD patients had pseudodementia features throughout a manic-depressive cycle (Table 2).

Psychomotor disturbances were common in our case series: five patients showed psychomotor retardation, three of which alternated with restlessness or agitation. Three patients also showed psychotic symptoms; in all cases, delusions had depressive content. Cognitive symptoms differed among patients. However, confusion and disorientation were typically prevalent in the early stages of pseudodementia, whereas memory alterations, executive dysfunction, and word-finding deficits could persist over time. Neuroimaging alterations were observed in all but one subject. White matter gliotic lesions have been found in the majority of the patients who have been described; however, these brain changes were not unequivocally associated with a negative outcome. Instead, all the patients who showed hypometabolism at the FDG-PET were found to have at least a slight impairment in functioning or a subjective decline in cognition at the end of the follow-up.

Symptoms lasted between 3 months and more than one year. Effective treatments included combined antidepressants in four cases, lithium salts in three cases, valproic acid in three cases, pregabalin or gabapentin in three cases, ECT in two cases, and clozapine and memantine in one case. Follow-up was variable, ranging between 6 months and 5 years. Variable outcomes emerged at the end of follow-up, independently from the diagnosis of unipolar or BD and early-onset or late-onset disorder. One patient who followed up for 5 years developed mild cognitive impairment, and one patient who followed up for more than 2 years had a progressive disability with cognitive, functional, and behavioral impairment. Subjective cognitive decline, reluctance to perform demanding tasks, and mild attention deficits were also, respectively, observed in two patients at follow-up.

## 4. Discussion

We described a case series of seven bipolar patients with pseudodementia. We also performed a critical review of the existing relevant literature, focusing on demographic features, family and personal histories, neuroimaging correlates, psychiatric and neurological symptoms, and the progression of cognitive symptoms.

In the elderly, different phases of BD can be accompanied by cognitive deficits [48] and may be mistakenly diagnosed as dementia. In a relevant proportion of these cases, it may be difficult to understand whether mood symptoms represent the initial stages of dementia. Indeed, BD can precede or arise in the context of neurodegenerative disorders [25,26,27].

Our sample was made up exclusively of female patients, limiting, in part, the generalizability of the results. In patients with mood disorders, a higher prevalence of pseudodementia in females than in male patients has also been found in previous research [49]. However, a contributing factor to the high proportion of females in our study may be the fact that all the inpatients in our sample were selected from a pool of subjects admitted to a female-only ward. In our patients, the age at onset of pseudodementia was higher than previously reported, and the large majority of our patients developed pseudodementia from the age of 65 onwards. To some extent, in our sample of subjects referred to a psychogeriatric outpatient service (65 years of age and older), the later onset may be largely due to selection bias. Pseudodementia seems to be more commonly observed during depressive episodes compared to (hypo)manic episodes [9,30,32,35,36,37]. In our case series, consistent with the previous literature, only one subject has had manic pseudodementia. However, other cases of pseudodementia occurring during a whole manic-depressive cycle and during a mixed manic episode have been previously described [31,40,42].

In line with the existing literature, psychomotor abnormalities were highly prevalent in our sample, especially in bipolar subjects. Psychomotor retardation is widely recognized as a marker for bipolarity in patients with depression [50]. As a matter of fact, all patients with BD and pseudodementia in our sample showed psychomotor retardation during the depressive phases. On the contrary, consistent with previous reports, in BD patients with pseudodementia in our case series, psychotic symptoms are not necessarily present [30,31,32,35,37,39,40,42,43,44].

According to the literature, ECT is the most commonly used treatment [29,32,36,44], and it has also been successfully employed in our unipolar and bipolar cases. Although lithium has been preferentially used in manic patients [31,38,39,41,42,45,46], in our case series, it was successfully employed in a BD patient with manic pseudodementia as well as in a BD patient with recurrent depressive pseudodementia. While the first one also received an antiepileptic and an antipsychotic, the second received an antiepileptic and dopaminergic treatment in combination with lithium. Despite the fact that the use of lithium in acute depressive episodes is debated, it is indicated as a treatment option for bipolar depression [51,52], and it is considered the first choice in the long-term prevention of BD episodes [53]. The remaining BD patient with an episode of depressive pseudodementia has been treated with a combination of antiepileptics and antidepressants. Although a case report of a BD patient with depressive pseudodementia treated with antidepressant monotherapy has been described [35], none of our BD patients received antidepressants in monotherapy due to the risk of antidepressant-induced mood switch and/or destabilization [53,54].

In most cases in our series, the duration of pseudodementia was 6 months or more in both unipolar and bipolar patients. In line with the literature, disorientation, especially in time, confusion, and executive dysfunctions emerged in the early stages of pseudodementia as the major cause of functional impairment in BD patients. According to Kiloh, who first described manic pseudodementia, “the grotesqueness of the responses should be sufficient to arouse suspicion, and the fact that for at least brief periods the patient reveals a startling appreciation of his surroundings should be sufficient to discount the impression of dementia” [2]. The criteria proposed by Wells may be useful both in the presence of manic and depressive symptoms [3]. Clinical features that can help identify patients with pseudodementia are their inability to perform even simple tasks, their tendency to emphasize cognitive disability, and an equally severe pattern of memory loss for recent and remote events [3].

All the patients in our sample underwent structural neuroimaging, and some of them also underwent functional neuroimaging. Consistent with previous reports on BD patients having pseudodementia [31,35,37,38,40,41,43], cortical atrophy was observed in a minority of our BD patients, and the same happened for subjects with unipolar depression, whereas white matter hyperintensities were more common. As it has been previously shown in the literature, the presence of cerebral abnormalities at structural neuroimaging is not evidence of a conclusive diagnosis of dementia [3]. In fact, mild cerebral atrophy has also been reported in elderly, not demented subjects [55]. Moreover, white matter changes seem to be broadly associated with mood disorders. White matter hyperintensities, indeed, are more prevalent in patients with BD and depression than in controls [56,57]. According to European guidelines, FDG-PET should be used as a supporting tool in the diagnosis of neurodegenerative diseases [58]. In our bipolar and unipolar subgroups, only a small percentage of patients had completed FDG-PET. Nevertheless, those in whom hypometabolism was discovered experienced functional, behavioral, and/or cognitive impairment during follow-up, providing evidence in favor of FDG-PET’s predictive utility for early identification of cognitive decline [59]. However, hypometabolism has been previously reported in a patient with depressive pseudodementia who showed a restoration of cerebral metabolism after antidepressant treatment [60]. This suggests that hypometabolism at FDG-PET cannot be regarded as distinctive of neurodegeneration since it can also be found in patients with depressive pseudodementia. On the other hand, the age at which mood disorders initially appeared did not seem to have a significant impact on the prognosis of either unipolar or bipolar patients; in fact, follow-up revealed functional or cognitive deficits in both patients with early and late-onset mood disorders (≥60 years). Although late-onset BD and late-onset depression have been linked to an increased risk of neurodegeneration in previous studies [13,26,61], an underlying vulnerability to cognitive decline cannot be excluded, even in patients with early-onset BD [62]. However, it is difficult to draw any conclusions on the impact of the different variables on the case prognosis due to the small sample size and differences in the duration of the follow-up. This study has limitations primarily because of the constraints of case reports and case series. Our findings, in fact, are not generalizable since causality cannot be inferred from uncontrolled observations. Moreover, due to the fact that case reports are not chosen from representative population samples, information on rates, ratios, incidences, or prevalences cannot be generated.

Importantly, up to 89% of unipolar depressive patients diagnosed with pseudodementia have been found to subsequently develop Alzheimer’s disease [8]. In addition, elderly patients with moderate to severe depression showing reversible cognitive impairment have a four-fold increased risk of subsequently developing dementia [49]. Several authors suggested renaming pseudodementia as ‘predementia’ or ‘pre-permanent dementia,’ underscoring the possible coexistence of depression and dementia and considering depressive pseudodementia as a prodromal or intermediate stage in the development of dementia [63,64,65].

Less is known about the outcome of manic-depressive pseudodementia, with evidence being limited to case reports or series. However, cognitive decline has been found in a substantial proportion of BD patients described in the reviewed studies, and further investigations are warranted.

## 5. Conclusions

Pseudodementia is likely to originate from heterogeneous mechanisms involving both affective state-related dysfunctions and neurodegenerative changes and their reciprocal interactions. Severe manic, mixed, and melancholic phases of BD could inherently present with prominent cognitive impairments, which could mimic dementia. On the other hand, patients with ongoing neurodegenerative processes could be more likely to develop late-life affective disorders or, if previously affected, to show more severe presentations. Finally, manic BD phases could prevent patients with otherwise high cognitive reserve from compensating for subclinical cognitive neurodegenerative decline.

Given the uncertain predictive validity, especially in patients with BD, the use of pseudodementia as a diagnostic entity could be misleading, and it should be better considered as a descriptive term whose prognostic value is yet to be defined. Modern technologies like genomic sequencing, detecting dementia-related biomarkers in biological fluids, and PET scans with tau and amyloid ligands may be able to shed light on the underlying causes of pseudodementia. Elucidating the nosology, mechanisms, and effective treatment of pseudodementia will require more investigation. In particular, further studies with larger patient samples, the adoption of proper diagnostic criteria, and a systematic follow-up method may be necessary.

## Figures and Tables

**Table 1:** Cases of patients with BD diagnosed with pseudodementia were reported in the literature.

Study	Age	Sex	Episode	Duration	Psychiatric Symptoms	Cognitive and Neurologic Symptoms	EEG	Neuroimaging	Treatment	BL MMSE	EP MMSE	FU	Outcomes
Chiles and Cohen, 1979 [41]	60	M	Manic	Several months	Hostility	Confusion, disorientation, memory loss	Marked diffused abnormalities	Atrophy	Lithium, Haloperidol	-	-	-	-
Cummings et al., 1980 [37]	56	M	Depressive	Few months	Flat affect, fixed expression, occasionally anger, uncooperativeness, restlessness	Tremor, paraphasia disorientation, memory loss, confusion, dyskinesia, hypomimia	Slow background rhythm with intermittent, scattered, 3- to 6-Hz slow activity	Cortical and central atrophy (CT)	Substantial medications decrease	-	-	1 y	Despite improvement in all areas, he had not regained his premorbid intellectual function
Cowdry and Goodwin, 1981 [31]	63	M	Manic depressive cycle	>3 y	Decreased sleep, increased talkativeness, activity, then withdrawal, anhedonia, guilt feelings, suicidal thoughts	Impairment of attention and short-term memory, confusion	-	Cortical atrophy	Lithium	-	-	25 m	Transient relapse treated with lithium
Koenigsberg, 1984 [46]	48	F	Manic						Lithium				
Thase and Reynolds, 1984 [42]	63	F	Manic	Weeks	Psychomotor agitation, withdrawal, neglect, decreased sleep, suicidal threats, pressured speech, irritability, affective lability, grandiose thoughts	Impairment of attention, comprehension, calculation, memory	Normal	Normal CT	Lithium	17–19	28	12 m	Transient relapse (depressive pseudodementia)
Bulbena and Berrios, 1986 [30]	75	M	Depressive	>6 m	-	-	-	-	-	-	-	15–47 m	Not demented
	66	F	Depressive	<6 m	Delusions, hallucinations	-	-	-	-	-	-	15–47 m	Dementia
	64	F	Depressive	>6 m	-	-	-	-	-	-	-	15–47 m	Not demented
	58	F	Depressive	<6 m	Delusions	-	-	-	-	-	-	15–47 m	Not demented
	51	F	Depressive	<6 m	-	-	-	-	-	-	-	15–47 m	Dementia
Casey and Fitzgerald, 1988 [38]	73	F	Manic	Several months	Psychomotor agitation, irritability, quarrelsomeness, insomnia, flight of ideas, hypersexuality	Disorientation to time, confusion, impaired attention and calculation, poor short-term memory	Mild diffuse slowing	Very mild cerebral atrophy, greatest in frontal regions, questionable old infarcts in the left periventricular area	Lithium	-	29	-	-
Wright and Silove, 1988 [39]	73	F	Manic	-	Increased activity, decreased sleep, excessive spending, flight of ideas, grandeur delusions, disinhibition, restlessness, fluctuating contradictory symptoms	Disorientation in time and place, poor concentration and short-term memory impairment, confabulation, perseveration	Normal	Normal CT	Lithium, then Fluphenazine	-	-	-	-
Kawai et al., 1990 [40]	67	M	Rapid cycling	7 y	Logorrhea, wandering, aggressiveness, quarrelsomeness, hoarding	Attention/memory disturbances, tremors, propulsive gait	Slight slowing of alpha activity	Slight sulcal enlargement and ventricular dilatation	-	-	-	3 y	Cognitive impairment
Sachdev et al., 1990 [9]	50	M	Manic	12 m	-	Cognitively impaired on neuropsychological evaluation, difficulty copying geometric figures	Normal	Normal CT or AEG	Lithium	-	-	11 y	Manic relapses; died of lithium toxicity
	49	F	Depressive	6 m	-	Cognitively impaired on neuropsychological evaluation	Intermittent slowing without diagnostic significance	Normal CT or AEG	-	-	-	12 y	Multiple relapses with remission
	63	F	Depressive	6 m	-	Cognitively impaired on neuropsychological evaluation	Normal	Normal CT or AEG	-	-	-	12 y	Multiple relapses with remission
	45	F	Depressive	12 m	-	Cognitively impaired on neuropsychological evaluation	Normal	Normal CT or AEG	-	-	-	12 y	Multiple relapses with remission
	56	F	Manic	4 m	-	Cognitively impaired on neuropsychological evaluation	Normal	Abnormal AEG	Lithium	-	-	12 y	Multiple relapses with good recovery
Parker and Austin, 1995 [36]	73	M	Depressive	Several months	Symptoms of melancholia, psychomotor retardation	-	Paroxysmal slow wave changes with a left-sided emphasis	Few small areas of punctate high signal (MRI). With the exception of some mild reduction in the posterior regions, overall, there is good intake (SPECT)	ECT	-	-	-	-
Banga et al., 2013 [44]	76	M	Mixed depression	3 m	Depressed mood, guilt feelings, suicidal gestures, decreased sleep, increased work activity, weight loss, paranoia	Fluctuating orientation levels, anomies, grossly disorganized speech, impairment in attention, memory, and executive function	Normal	Moderate hypoperfusion within the left parietal lobe/temporoparietal cortex, mild hypoperfusion within the right parietal lobe/posterior temporoparietal cortex and the frontal lobe (SPECT)	Venlafaxine, Mirtazapine, Aripiprazole	13–23	30	7 y	Two relapses, one treated with ECT
Rapinesi et al., 2013 [32]	71	F	Depressive	-	Severe psychomotor retardation, insomnia	-	Normal	-	ECT	5	23	-	-
	65	F	Depressive	-	Psychomotor agitation, anxiety	-	Normal	-	ECT	9	17	-	-
	79	F	Depressive	-	Behavioral alterations, delusions, anxiety	-	Normal	-	ECT	12	27	-	-
	65	F	Depressive	-	Severe psychomotor retardation, fatigue, insomnia	-	Diffuse slow waves	-	ECT	14	22	-	-
	79	F	Depressive	-	Behavioral alterations, apathy, weight loss	-	Minor irregularity	-	ECT	17	25	-	-
	75	M	Depressive	-	Behavioral alterations, anxiety	-	Normal	-	ECT	17	26	-	-
	69	F	Depressive	-	Psychomotor agitation, behavioral alterations	-	Normal	-	ECT	18	28	-	-
	66	F	Depressive	-	Irritability, insomnia, impulsiveness	-	Normal	-	ECT	20	24	-	-
Woudstra et al., 2014 [35]	60	F	Depressive	2 y	Nihilistic hallucinations, delusions, anxiety	Orientation, short-term memory, executive function deficits, apraxia, dyskinesia	-	Global cortical atrophy, mild atrophy of the hippocampus, especially on the left. Subcortical hyperintensities. Hypometabolism of the right parietal/temporal cortex and cerebellar hemisphere (FDG-PET)	Nortriptyline	11–12	29	-	-
Ciappolino and Orsenigo, 2018 [43]	71	M	Mixed	Several months	Restlessness, irritability, talkativeness, sleeplessness, guilt delusions, paranoia	Confusion, fluctuating orientation, short-term/attention deficits, disorganized in speech/thinking	Normal	Prominent cerebral sulci, ventriculomegaly, ischemic lesions in both frontal regions, and periventricular leukoaraiosis (CT). Normal uptake, except for an area of hypocaptation (FDG-PET)	Quetiapine	17–24	29	-	-

Abbreviations: AD: Alzheimer’s disease; AEG: air encephalogram; FDG-PET: 18-Fluorodeoxyglucose Positron Emission Tomography; BL: baseline; CSF: cerebrospinal fluid; CT: computed tomography; ECT: electroconvulsive therapy; EEG: electroencephalogram; EP: end point; F: female; FU: follow-up; M: male; MMSE: Mini-Mental State Examination; m: months; SPECT: single-photon emission computed tomography; y: years.

**Table 2:** Cases of patients with mood disorders diagnosed with pseudodementia who were referred to our Psychiatric Department.

	Late-Onset, Single-Episode Depressive Pseudodementia	Depressive Pseudodementia in a Patient with Late-Onset Recurrent MDD	Depressive Pseudodementia in a Patient with Recurrent MDD and OCD Comorbidity	Depressive Pseudodementia in a Patient with BD Type 2	Depressive Pseudodementia with Psychosis in a Patient with BD Type 2 and OCD Comorbidity	Recurrent Depressive Pseudodementia in a Patient with BD Type 2	Manic-Depressive Pseudodementia in a Patient with Late-Onset BD Type 1
Age	74	76	60	69	58	81	73
Age of onset mood disorder	72	67	30	20	14	24	64
Psychiatric comorbidity	-	-	OCD	Panic disorder	OCD	-	Social anxiety
Somatic comorbidity	Diabetes mellitus type 2, hypertension, osteoporosis, grade I cystocele	-	Hypertension	Diabetes mellitus type 2, hypertension	Hypertension, hypercholesterolemia, nephrolithiasis, obesity	Dyslipidemia, thrombocytosis	Hypertension, hypothyroidism
Duration of pseudodementia	1 y	<6 m	>1 y	Almost 1 y	3 m	<6 m	5 m
Psychiatric symptoms	Depressed mood, anxiety, insomnia, hyporexia	Depressed mood, internal tension, panic attacks, lability, somatizations, insomnia	Depressed mood, anxiety, obsessive thoughts, checking rituals, verbal perseveration	Low mood, lack of energy, hypersomnia, loss of interest, hopelessness, anxiety, behavioral impairments, agoraphobia	Depressive mood, anxiety, guilt ideas, obsessive doubts, cleaning rituals, insomnia, refusal to eat and drink	Depressed mood, obsessive thoughts, checking rituals, holothymic themes, anxiety, pragmatic inhibition, insomnia	Irritability, physical/verbal aggressiveness followed by clinophilia, oppositional/disinhibited behaviors, coprolalia, perseveration, verbal aggression, refusal to eat, drink and take medications
Psychomotor features	Psychomotor slowdown alternated with restlessness	-	Psychomotor retardation alternated with agitation	Psychomotor retardation, hypomimia, rigidity and bradykinesia	Psychomotor slowdown alternated with episodes of agitation and restlessness, especially during night	Psychomotor retardation, hand resting tremors	Increased energy followed by psychomotor slowdown, clinophilia, negativism
Cognitive and neurologic symptoms	Scarce overall functioning, difficulties with taking care of her personal needs	Memory deficits, difficulties with doing regular tasks	Disorientation in time, severe attention deficits, reduction of autonomy, recall difficulties, and word-finding deficits	Deficits in verbal learning, selective and divided attention, several episodes of confusion	Mental confusion, disorientation in time, and marked difficulties in performing simple tasks	Disorientation in time, deficits in short-term memory, executive deficits with difficulties doing housework	Disorientation, confusion, confabulation, grossly memory deficits, and difficulties with taking care of her personal needs
Psychosis	Suspiciousness, theme of ruin, somatic delusions	-	-	-	Somatic delusion	Feelings of guilt and incurability and themes of ruin	-
Neuroimaging	Gliosis resulting from chronic vascular encephalopathy (CT)	Modest hypodensity of the periventricular white matter (CT)	Slight enlargement of periencephalic spaces at the vertex, areas of hyperintensities in the corona radiata and orbital cortex (MRI); mild global hypometabolism in the cortex, mostly in frontal areas (FDG-PET)	Areas of hyperintensity in the frontal subcortical white matter (MRI); no metabolic alterations (FDG-PET)	Normal (CT)	Leukoencephalopathy with lacunar lesions in the subcortical white matter and a small meningioma in the frontal right lobe (MRI); hypometabolism in the frontotemporal left lobe (FDG-PET)	Predominant left hemisphere atrophy, with a consensual enlargement of the ventricular system (MRI); moderate hypometabolism of the left hemisphere (FDG-PET)
Treatment	13 ECT session	Paroxetine 20 mg/day, amitriptyline 50 mg/day, trimipramine 20 mg/day and pregabalin 150 mg/day	Valproic acid 750 mg/day, trazodone 50 mg/day, and delorazepam 1 mg/day. Then valproic acid, fluvoxamine 300 mg/day, clomipramine 20 mg/day, lithium carbonate 450 mg/day, quetiapine 125 mg/day	Paroxetine 10 mg/day, clomipramine 50 mg/day, trimipramine 20 mg/day, pregabalin 100 mg/day	8 ECT session	Lithium carbonate, gabapentin, amantadine (1st episode); valproic acid 600 mg/day and mianserin 30 mg/day (2nd episode)	Valproic acid 1000 mg/day, lithium salts 450 mg/day, clozapine 75 mg/day, and memantine 10 mg/day
BL MMSE	26	25	21	21	24	14 (2nd)	Not administrable
EP MMSE	27	20	30	29	30	15 (2nd)	29/30
FU	4 y	5 y	2 y	4 y	>1 y	>2 y	2 y
Outcomes	Not demented	Mild Cognitive Impairment	Not demented but reluctant to perform demanding tasks	Not demented	Not demented	Progressive cognitive, behavioral, and functional impairment	Not demented, subjective cognitive deficits

Abbreviations: BD: bipolar disorder; FDG-PET: 18-Fluorodeoxyglucose Positron Emission Tomography; BL: baseline; CT: computed tomography; ECT: electroconvulsive therapy; EP: end point; FU: follow-up; MDD: major depressive disorder; MMSE: Mini-Mental State Examination; MRI: magnetic resonance imaging; m: months; OCD: obsessive–compulsive disorder; y: years.

## Data Availability

Not applicable.

## References

[1] Burns A., Jolley D. (2015). Pseudodementia: History, mystery and positivity. Troublesome Disguises: Managing Challenging Disorders in Psychiatry.

[2] Kiloh L.G. (1961). Pseudo-dementia. Acta Psychiatr. Scand..

[3] Wells C.E. (1979). Pseudodementia. Am. J. Psychiatry.

[4] Copeland J.R., Davidson I.A., Dewey M.E., Gilmore C., Larkin B.A., McWilliam C., Saunders P.A., Scott A., Sharma V., Sullivan C. (1992). Alzheimer’s disease, other dementias, depression and pseudodementia: Prevalence, incidence and three-year outcome in Liverpool. Br. J. Psychiatry.

[5] Clarfield A.M. (1988). The reversible dementias: Do they reverse?. Ann. Intern. Med..

[6] Clarfield A.M. (2003). The decreasing prevalence of reversible dementias: An updated meta-analysis. Arch. Intern. Med..

[7] Connors M.H., Quinto L., Brodaty H. (2019). Longitudinal outcomes of patients with pseudodementia: A systematic review. Psychol. Med..

[8] Kral V.A., Emery O.B. (1989). Long-Term Follow-up of Depressive Pseudodementia of the Aged. Can. J. Psychiatry.

[9] Sachdev P.S., Smith J.S., Angus-Lepan H., Rodriguez P. (1990). Pseudodementia twelve years on. J. Neurol. Neurosurg. Psychiatry.

[10] Pearlson G.D., Rabins P.V., Kim W.S., Speedie L.J., Moberg P.J., Burns A., Bascom M.J. (1989). Structural brain CT changes and cognitive deficits in elderly depressives with and without reversible dementia (‘pseudodementia’). Psychol. Med..

[11] Mahendra B. (1983). “Pseudodementia” a misleading and illogical concept. Br. J. Psychiatry.

[12] Leyhe T., Reynolds C.F., Melcher T., Linnemann C., Klöppel S., Blennow K., Zetterberg H., Dubois B., Lista S., Hampel H. (2017). A common challenge in older adults: Classification, overlap, and therapy of depression and dementia. Alzheimers Dement..

[13] Cherbuin N., Kim S., Anstey K.J. (2015). Dementia risk estimates associated with measures of depression: A systematic review and meta-analysis. BMJ Open.

[14] Elefante C., Brancati G.E., Petrucci A., Gemmellaro T., Toni C., Lattanzi L., Perugi G. (2022). Risk of conversion to bipolar disorder in patients with late-onset major depression. Int. Clin. Psychopharmacol..

[15] do Nascimento K.K.F., Silva K.P., Malloy-Diniz L.F., Butters M.A., Diniz B.S. (2015). Plasma and cerebrospinal fluid amyloid-β levels in late-life depression: A systematic review and meta-analysis. J. Psychiatr. Res..

[16] Brodaty H., Connors M.H. (2020). Pseudodementia, pseudo-pseudodementia, and pseudodepression. Alzheimer’s Dement..

[17] Ghaemi S.N., Sachs G.S., Chiou A.M., Pandurangi A.K., Goodwin K. (1999). Is bipolar disorder still underdiagnosed? Are antidepressants overutilized?. J. Affect. Disord..

[18] Ghaemi S.N., Boiman E.E., Goodwin F.K. (2000). Diagnosing bipolar disorder and the effect of antidepressants: A naturalistic study. J. Clin. Psychiatry.

[19] Loranger A.W., Levine P.M. (1978). Age at onset of bipolar affective illness. Arch. Gen. Psychiatry.

[20] Taylor M.A., Abrams R. (1973). The phenomenology of mania. A new look at some old patients. Arch. Gen. Psychiatry.

[21] Depp C.A., Lindamer L.A., Folsom D.P., Gilmer T., Hough R.L., Garcia P., Jeste D.V. (2005). Differences in clinical features and mental health service use in bipolar disorder across the lifespan. Am. J. Geriatr. Psychiatry Off. J. Am. Assoc. Geriatr. Psychiatry.

[22] Kessler R.C., Berglund P., Demler O., Jin R., Merikangas K.R., Walters E.E. (2005). Lifetime prevalence and age-of-onset distributions of DSM-IV disorders in the national comorbidity survey replication. Arch. Gen. Psychiatry.

[23] Depp C.A., Jeste D. (2004). V Bipolar disorder in older adults: A critical review. Bipolar Disord..

[24] Dols A., Kupka R.W., van Lammeren A., Beekman A.T., Sajatovic M., Stek M.L. (2014). The prevalence of late-life mania: A review. Bipolar Disord..

[25] Bacciardi S., Elefante C., Brancati G.E., Mazzucchi S., Del Prete E., Frosini D., Maremmani I., Lattanzi L., Ceravolo R., Bonuccelli U. (2022). Bipolar Spectrum disorders in Parkinson’s disease: A systematic evaluation. CNS Spectr..

[26] Elefante C., Brancati G.E., Torrigiani S., Amadori S., Ricciardulli S., Pistolesi G., Lattanzi L., Perugi G. (2023). Bipolar Disorder and Manic-like Symptoms in Alzheimer’s, Vascular and Frontotemporal Dementia: A Systematic Review. Curr. Neuropharmacol..

[27] Ng B., Camacho A., Lara D.R., Brunstein M.G., Pinto O.C., Akiskal H.S. (2008). A case series on the hypothesized connection between dementia and bipolar spectrum disorders: Bipolar type VI?. J. Affect. Disord..

[28] Page M.J., McKenzie J.E., Bossuyt P.M., Boutron I., Hoffmann T.C., Mulrow C.D., Shamseer L., Tetzlaff J.M., Akl E.A., Brennan S.E. (2021). The PRISMA 2020 statement: An updated guideline for reporting systematic reviews. BMJ.

[29] Allen R.M. (1982). Pseudodementia and ECT. Biol. Psychiatry.

[30] Bulbena A., Berrios G.E. (1986). Pseudodementia: Facts and Figures. Br. J. Psychiatry.

[31] Cowdry R.W., Goodwin F.K. (1981). Dementia of bipolar illness: Diagnosis and response to lithium. Am. J. Psychiatry.

[32] Rapinesi C., Serata D., Del Casale A., Kotzalidis G.D., Mazzarini L., Fensore C., Carbonetti P., Scatena P., Capezzuto S., Moscati F.M. (2013). Depressive pseudodementia in the elderly: Effectiveness of electroconvulsive therapy. Int. J. Geriatr. Psychiatry.

[33] Reid S.D. (2000). Pseudodementia in a twenty-one-year-old with bipolar disorder and vitamin B12 and folate deficiency. West Indian Med. J..

[34] Mecê A.M., Abreu V.C., Lamas G.M., Tacla R.D.R., Minekawa T.B., Ramos C.D., Balthazar M.L.F. (2022). Lithium Intoxication as a cause of reversible dementia mimicking FDG PET features of Alzheimer’s disease. Dement. Neuropsychol..

[35] Woudstra F.H., van de Poel-Mustafayeva A.T., van der Ploeg M.V., de Vries J.J., van der Lek R.F.R., Izaks G.J. (2014). Symptoms mimicking dementia in a 60-year-old woman with bipolar disorder: A case report. BMC Res. Notes.

[36] Parker G., Austin M.-P. (1995). A clinical perspective on SPECT. Aust. N. Z. J. Psychiatry.

[37] Cummings J., Benson D.F., Loverme S. (1980). Reversible Dementia: Illustrative Cases, Definition, and Review. JAMA J. Am. Med. Assoc..

[38] Casey D.A., Fitzgerald B.A. (1988). Mania and pseudodementia. J. Clin. Psychiatry.

[39] Wright J.M., Silove D. (1988). Pseudodementia in schizophrenia and mania. Aust. N. Z. J. Psychiatry.

[40] Kawai M., Miyamoto K., Miyamoto M. (1990). A Case of Elderly Manic-Depression with Dementia-Like Symptoms in a Manic Phase. Clin. Gerontol..

[41] Chiles J.A., Cohen D. (1979). Pseudodementia and Mania. J. Nerv. Ment. Dis..

[42] Thase M.E., Reynolds C.F. (1984). Manic pseudodementia. Psychosomatics.

[43] Ciappolino V., Orsenigo G. (2019). Pseudodementia: A case report on the connection between dementia and bipolar spectrum disorders. Clinical Cases in Psychiatry: Integrating Translational Neuroscience Approaches.

[44] Banga A., Gyurmey T., Matuskey D., Connor D.F., Kaplan R.F., Steffens D.C. (2013). Late-life onset bipolar disorder presenting as a case of pseudo-dementia: A case discussion and review of literature. Yale J. Biol. Med..

[45] Smith J.S., Kiloh L.G. (1981). The investigation of dementia: Results in 200 consecutive admissions. Lancet.

[46] Koenigsberg H.W. (1984). Manic pseudodementia: Case report. J. Clin. Psychiatry.

[47] Petrides G., Fink M. (1996). The “half-age” stimulation strategy for ECT dosing. Convuls. Ther..

[48] Lima I.M.M., Peckham A.D., Johnson S.L. (2018). Cognitive deficits in bipolar disorders: Implications for emotion. Clin. Psychol. Rev..

[49] Sáez-Fonseca J.A., Lee L., Walker Z. (2007). Long-term outcome of depressive pseudodementia in the elderly. J. Affect. Disord..

[50] Calugi S., Cassano G.B., Litta A., Rucci P., Benvenuti A., Miniati M., Lattanzi L., Mantua V., Lombardi V., Fagiolini A. (2011). Does psychomotor retardation define a clinically relevant phenotype of unipolar depression?. J. Affect. Disord..

[51] Yatham L.N., Kennedy S.H., Parikh S.V., Schaffer A., Bond D.J., Frey B.N., Sharma V., Goldstein B.I., Rej S., Beaulieu S. (2018). Canadian Network for Mood and Anxiety Treatments (CANMAT) and International Society for Bipolar Disorders (ISBD) 2018 guidelines for the management of patients with bipolar disorder. Bipolar Disord..

[52] Haeberle A., Greil W., Russmann S., Grohmann R. (2012). Mono- and combination drug therapies in hospitalized patients with bipolar depression. Data from the European drug surveillance program AMSP. BMC Psychiatry.

[53] Sani G., Perugi G., Tondo L. (2017). Treatment of Bipolar Disorder in a Lifetime Perspective: Is Lithium Still the Best Choice?. Clin. Drug Investig..

[54] Viktorin A., Lichtenstein P., Thase M.E., Larsson H., Lundholm C., Magnusson P.K.E., Landén M. (2014). The risk of switch to mania in patients with bipolar disorder during treatment with an antidepressant alone and in combination with a mood stabilizer. Am. J. Psychiatry.

[55] Tomlinson B.E., Blessed G., Roth M. (1968). Observations on the brains of non-demented old people. J. Neurol. Sci..

[56] Herrmann L.L., Le Masurier M., Ebmeier K.P. (2008). White matter hyperintensities in late life depression: A systematic review. J. Neurol. Neurosurg. Psychiatry.

[57] Tighe S.K., Reading S.A., Rivkin P., Caffo B., Schweizer B., Pearlson G., Potash J.B., Depaulo J.R., Bassett S.S. (2012). Total white matter hyperintensity volume in bipolar disorder patients and their healthy relatives. Bipolar Disord..

[58] Nobili F., Arbizu J., Bouwman F., Drzezga A., Agosta F., Nestor P., Walker Z., Boccardi M., EANM-EAN Task Force for the Prescription of FDG-PET for Dementing Neurodegenerative Disorders (2018). European Association of Nuclear Medicine and European Academy of Neurology recommendations for the use of brain ^18^F-fluorodeoxyglucose positron emission tomography in neurodegenerative cognitive impairment and dementia: Delphi consensus. Eur. J. Neurol..

[59] Jagust W., Mungas D., Decarli C. (2007). What does fluorodeoxyglucose PET imaging add to a clinical diagnosis of dementia?. Neurology.

[60] Pozzi F.E., Licciardo D., Musarra M., Jonghi-Lavarini L., Crivellaro C., Basso G., Appollonio I., Ferrarese C. (2022). Depressive Pseudodementia with Reversible AD-like Brain Hypometabolism: A Case Report and a Review of the Literature. J. Pers. Med..

[61] Heser K., Tebarth F., Wiese B., Eisele M., Bickel H., Köhler M., Mösch E., Weyerer S., Werle J., König H.-H. (2013). Age of major depression onset, depressive symptoms, and risk for subsequent dementia: Results of the German study on Ageing, Cognition, and Dementia in Primary Care Patients (AgeCoDe). Psychol. Med..

[62] Almeida O.P., Fenner S. (2002). Bipolar Disorder: Similarities and Differences between Patients with Illness Onset before and after 65 Years of Age. Int. Psychogeriatr..

[63] Reifler B.V. (2000). A Case of Mistaken Identity: Pseudodementia Is Really Predementia. J. Am. Geriatr. Soc..

[64] Emery V., Oxman T. (2003). Depressive dementia: A “prepermanent intermediate-stage dementia” in a long-term disease course of permanent dementia?. Dementia: Presentations, Differential Diagnosis and the Nosology.

[65] Reifler B.V., Larson E., Hanley R. (1982). Coexistence of cognitive impairment and depression in geriatric outpatients. Am. J. Psychiatry.

